# Burn Management in a Patient With Acute Exacerbation of Comorbid Systemic Lupus Erythematosus

**DOI:** 10.7759/cureus.43385

**Published:** 2023-08-12

**Authors:** Bayli P Davis, Alan Pang, Hussain R Abidi, John Griswold

**Affiliations:** 1 Department of Surgery, Texas Tech University Health Sciences Center, Lubbock, USA; 2 Department of Dermatology, Texas Tech University Health Sciences Center, Lubbock, USA

**Keywords:** wound healing, immunosuppression, burn, lupus, sle

## Abstract

A 28-year-old female presented to the burn unit with 2% total body surface area second-degree burns to the right flank and right breast after accidentally spilling coffee on herself while hospitalized for an acute exacerbation of systemic lupus erythematosus (SLE) in the form of neuromyelitis optica spectrum disorder. We document her inpatient management, which was challenging because of the contradictory relationship between typical management of SLE exacerbations (i.e., immunosuppressive medication regimens) and the body’s post-burn healing process, which is inherently inflammatory in nature. Even with a high-dose immunosuppressive medication regimen, our patient’s second-degree burns healed with non-operative management without significant adverse events. Adding to a small yet growing body of literature addressing the clinical presentation and management of burn wounds in the setting of an acute SLE exacerbation, our case suggests that clinicians must carefully weigh the risks of surgical intervention with those of non-operative management when approaching burn care during an acute rheumatologic disease flare up.

## Introduction

This article was previously presented as a poster at the 21st Congress of the International Society for Burn Injuries in Guadalajara, Mexico, in August 2022.

Systemic lupus erythematosus (SLE) is an autoimmune disorder in which the body elicits an immune response against its own tissues, resulting in widespread inflammation and multi-organ damage [1]. This disease affects numerous tissues including the kidneys, brain and central nervous system, joints, blood vessels, lungs, and heart. Clinical manifestations of this disease include rash, arthritis, serositis, hematologic disorders, mucosal ulcers, renal disease, and neurologic disorders [2].

The underlying pathophysiology behind SLE is complex, but it has been shown that an aberrant adaptive immune response involving B and T lymphocyte activation and subsequent autoantibody-mediated tissue damage is the major process contributing to widespread inflammation [3]. Because of the inherent inflammatory nature of SLE, therapeutic strategies are primarily centered around suppressing the pathological immune response with immunosuppressive pharmaceutical agents such as, but not limited to, corticosteroids, monoclonal antibodies, hydroxychloroquine, azathioprine, mycophenolate mofetil, cyclophosphamide, and methotrexate [4-6].

Like SLE, the physiological response to thermal burn injury relies heavily on inflammation. The healing process begins with the release of histamine, free radicals, and inflammatory cytokines, which ultimately vasodilate blood vessels and attract immune cells that phagocytose necrotic debris and release growth factors that stimulate cell proliferation and ultimate regeneration of tissue [7]. Thus, the elicitation of an inflammatory cascade of events is paramount to the body’s response to tissue injury.

Importantly, immunosuppressive drug regimens used to treat SLE, such as long-term systemic corticosteroids, methotrexate, mycophenolate mofetil, azathioprine, and drugs interfering with the Interleukin-2 mediated immune response (e.g., voclosporin), as well as the presence of SLE per se, counteract and impair the wound healing process [8-13]. This potentially complicates the management of burn patients with comorbid SLE and necessitates special consideration on how to approach burn management in this unique population.

## Case presentation

A 28-year-old female with a past medical history of SLE, stage 4 lupus nephritis, renal vein thrombosis, fibromyalgia, hypertension, and recent COVID-19 infection was admitted to the hospital for neurological manifestations in the setting of lupus. She initially presented to the emergency department with complaints of right lower extremity numbness, pain, and weakness with associated bladder and bowel incontinence. Head CT scan showed unchanged hypodensities and calcification of parenchyma. MRI of the spine showed multiple enhancing lesions in the brainstem, cerebellum, cervical spine, and thoracic spine. MRI of the brain showed subtle T2 hyperintensity in the left optic nerve. Lumbar puncture was negative for infection. She was subsequently found to be positive for AQP4-IgG and was diagnosed with neuromyelitis optica spectrum disorder, a condition frequently seen in association with autoimmune conditions such as SLE. Her neurological symptoms failed to improve initially with methylprednisolone monotherapy. Symptom resolution was ultimately achieved after a seven-day course of methylprednisolone followed by a five-day course of intravenous immunoglobulin (IVIG). Her hospital course was complicated by several factors. She developed acute kidney injury (AKI) that was attributed to her known stage 4 lupus nephritis; this was initially treated with aggressive diuresis as well as the resumption of her daily home (maintenance) regimen consisting of mycophenolate mofetil and hydroxychloroquine, as well as methylprednisolone in place of her usual outpatient prednisone. In addition, during her hospital course, she developed gastrointestinal bleeding for which she was started on pantoprazole, discontinued apixaban, and administered two units of packed red blood cells.

**Figure 1:**
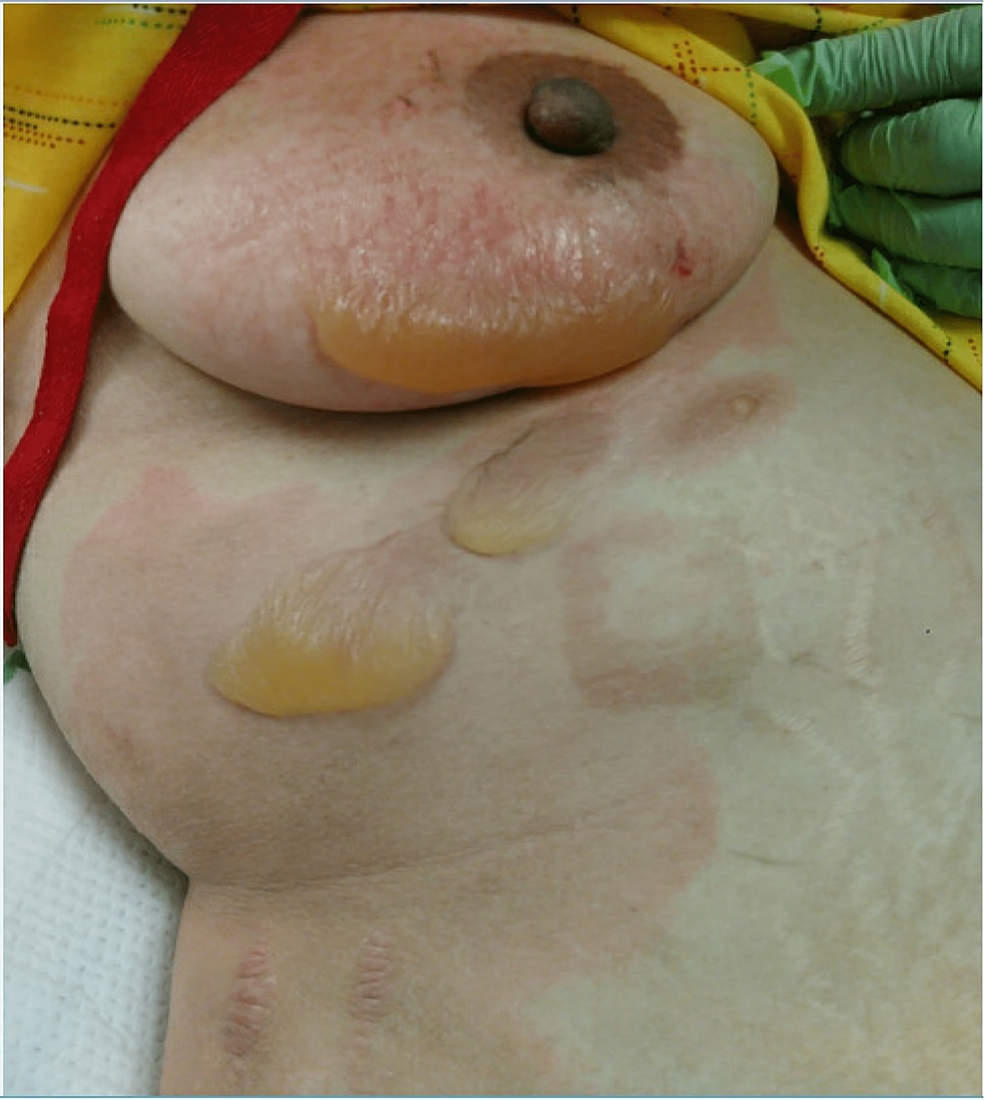
Burn wounds on initial presentation to the burn unit

The burn unit was consulted for this patient on hospital day 15 after she accidentally spilled coffee on herself while in the hospital. She was found to have 2% total body surface area and second-degree scald burns to the right flank and right breast. Following the initial assessment by our care team, her burn wounds were cleaned and dressed; PluroGel, Aquaphor, and chlorhexidine (CHG) were applied to the wounds. On post-burn day 1, the rheumatological management plan was to continue mycophenolate mofetil 1000 mg oral twice daily, hydroxychloroquine 200 mg oral twice daily, and methylprednisolone 125 mg IV twice daily. Due to the incomplete resolution of her AKI following diuresis and resumption of her home medications, the decision was made on post-burn day 3 to escalate immunosuppressive therapy by adding cyclophosphamide with mesna to her inpatient rheumatologic regimen. In addition to ongoing surveillance and correspondence by rheumatology, nephrology, neurology, and the burn team during her inpatient course thus far, subsequent consults were made to the hospital's nutritionists, physical therapy department, and wound care teams. Regarding burn management, the patient underwent daily wound cleansing with CHG, mechanical debridement, and dressing change with the application of PluroGel, Aquaphor/Adaptic, dry gauze, and tape. Pain management throughout her hospital course consisted of hydrocodone-acetaminophen, which was deescalated as tolerated by the patient. The patient’s burn wounds on the initial presentation are shown in Figure 1.

**Figure 2:**
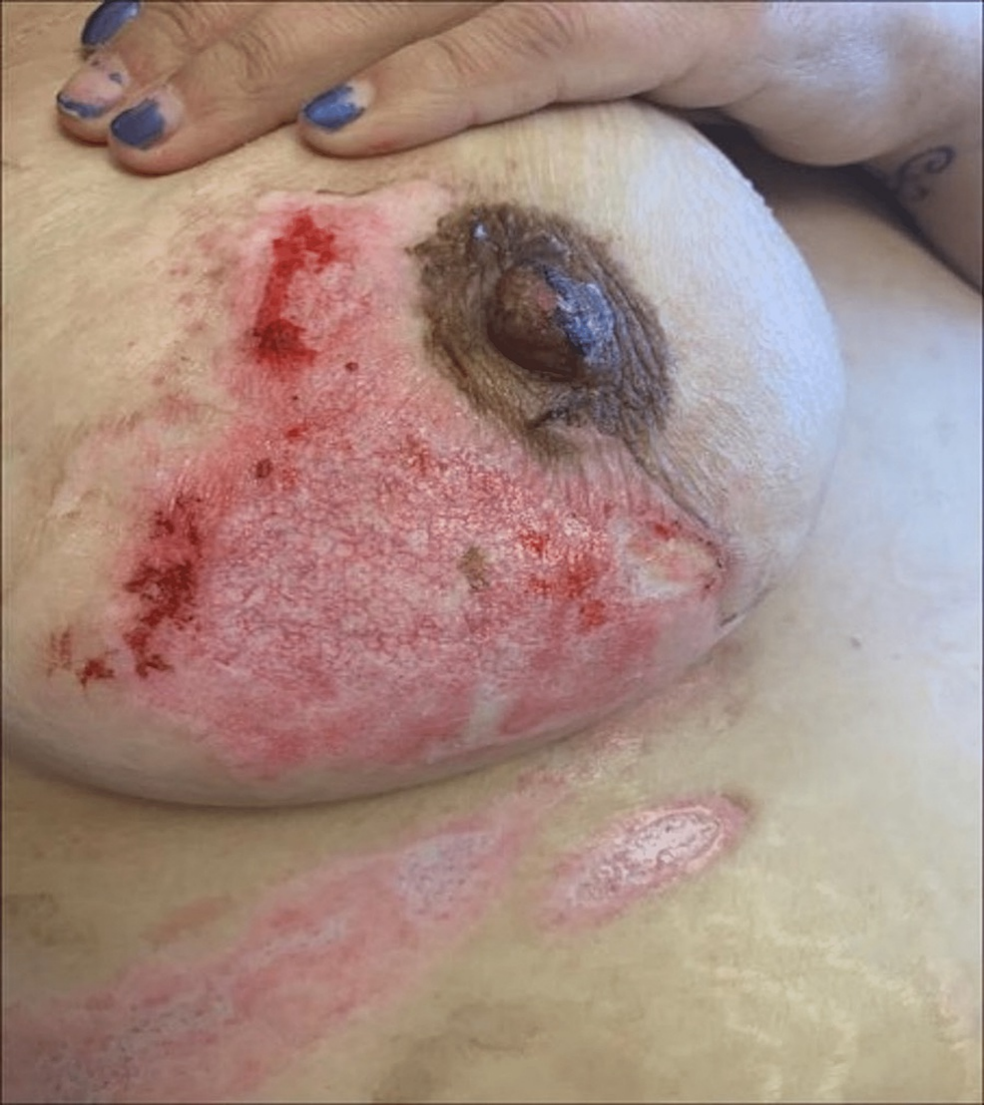
Wounds on post-burn day 12

**Figure 3:**
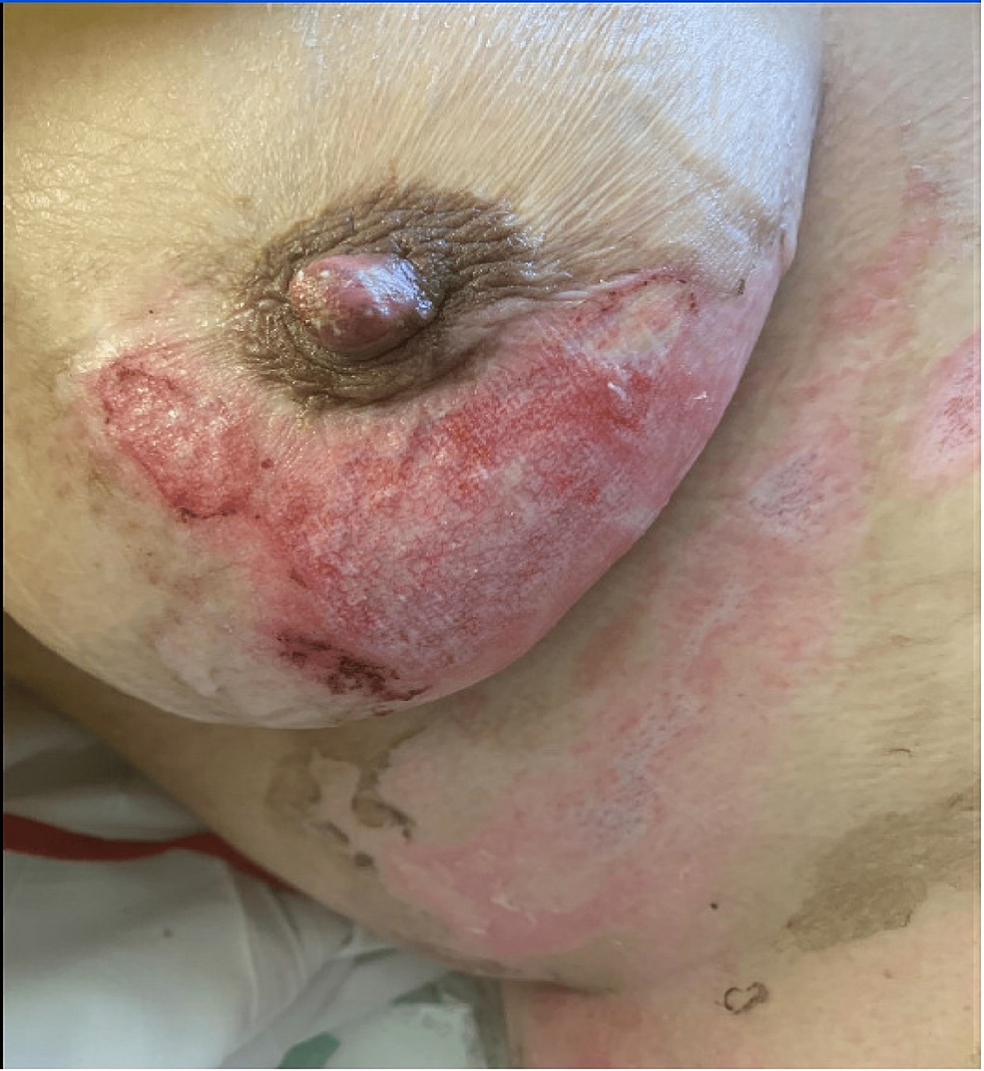
Wounds on post-burn day 13

On post-burn day 2, the patient’s wounds were noted to be healing very slowly. By post-burn day 6, the wounds still had a moderate amount of nonviable tissue (NVT). Starting on post-burn day 7, Sulfamylon was applied and the use of PluroGel was discontinued. By post-burn day 8, there was an additional 10% conversion of burn wounds to NVT. On post-burn day 10, the topical was changed back to PluroGel, which was applied to all areas of burns with NVT. Nivea cream was applied to all healed burns at this point. Wounds on post-burn days 12 and 13 are shown in Figures 2-3, respectively; the majority of granulation tissue had formed by this point.

**Figure 4:**
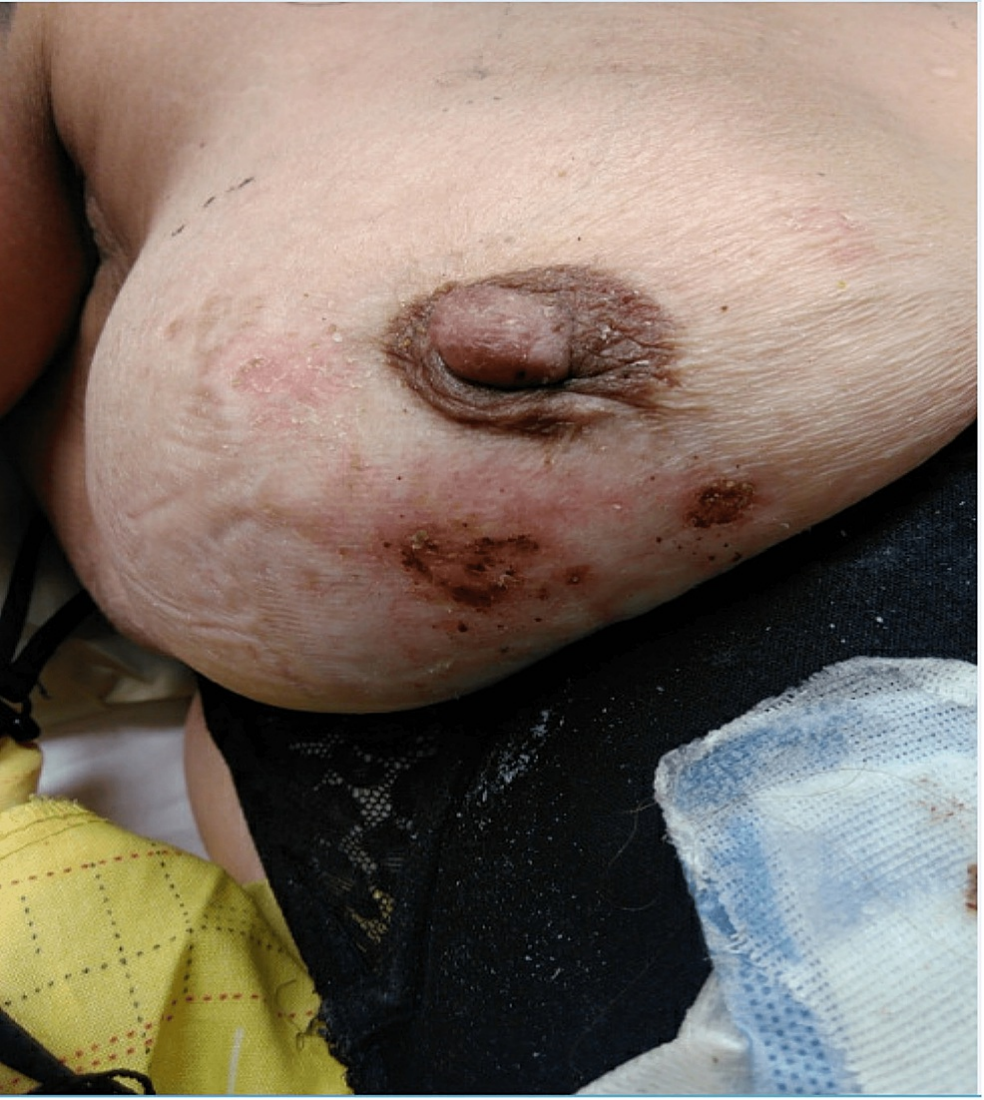
Wounds upon readmission at post-burn day 80 for a subsequent SLE exacerbation

By post-burn day 15, most wound tissue was healed except for the right breast. The possibility of split-thickness skin grafting was considered at this time but ultimately was not needed. The patient was discharged on post-burn day 12 with instructions to apply a debriding surfactant daily to the wounds and follow up as an outpatient. She was unfortunately lost to follow-up and re-evaluated on subsequent admission for another exacerbation of her lupus several months later. Figure 4 shows the burn wounds on her second admission, post-burn day 80.

## Discussion

There is a considerable lack of research concerning burn management in the setting of a severe exacerbation of SLE requiring a multi-agent immunosuppressive regimen. In one of the few studies to assess burn management in patients with concomitant autoimmune rheumatic diseases, including SLE, 2.7% of subjects that had a history of autoimmune rheumatic disease were more likely to require additional therapeutic interventions such as renal replacement and enteral nutrition [12]. In this patient, therapeutic measures included placing the patient on a renal diet, pain management for burn and nephritis-associated pain, anticoagulation due to renal vein thrombosis, IVIG and steroids to stifle an overactive immune response, and management of complications due to GI bleeding and leukopenia. Notably, no standards exist to guide the treatment approach to burn injury in patients with autoimmune conditions such as SLE, let alone in the setting of a severe acute SLE exacerbation.

Regarding burn management, our patient was observed to have a slightly slowed wound healing process superimposed on rheumatologic management with an immunosuppressive medication regimen she was started on for suspected neuromyelitis optica. This implied that the two treatment approaches, one addressing her neurologic presentation and one addressing her dermatologic presentation, counteracted one another. As the patient’s body elicited a pro-inflammatory response to her burn wounds, her anti-inflammatory medication regimen served to tone down this process, possibly delaying tissue repair and regeneration. In this patient’s hospital course, this suggests that the treatment approach required considerable balance due to the risk of hindering the resolution of one problem by treating another.

What warrants further research is if a superior treatment approach, one that caters to both autoimmune disease manifestations exacerbations as well as burn injuries, exists. The multisystemic nature of SLE demands consultation by a diverse team of physicians including rheumatology, nephrology, neurology, and many more. Currently, the management of SLE depends on disease severity and manifestations, with hydroxychloroquine and immunosuppressive agents playing central roles in long-term treatment [13]. However, immunomodulatory agents can play a role in slow wound healing. In the setting of burn injuries, concomitant management of SLE may place more importance on agents that are not anti-inflammatory. Currently, these include plasma exchange, dietary therapy, and behavioral changes, such as avoiding direct sunlight. On the other hand, further research into alternative strategies to manage burn wounds that permit the continuation of immunosuppressive agents would be beneficial to these patients.

As noted previously, split-thickness skin grafting was considered at one point by the patient’s burn care team. While skin grafting is a frequent approach in typical burn cases, special considerations are needed when considering such procedures for patients with comorbid autoimmune diseases, such as the patient at hand. Immunosuppressive medications, such as corticosteroids, are known to significantly increase the risk of certain post-operative wound complications, such as wound site infection and dehiscence [14]. A study from 2009 demonstrated that lupus-prone mice were significantly more likely to reject skin isografts compared to mice without features of autoimmune disease [15]. Though this study involved mice and not humans, it does suggest that graft take may be sub-optimal in patients with comorbid SLE. Special consideration is warranted in cases where skin grafting is considered as part of the treatment approach to burn injury in a patient with SLE. It is also important to highlight and contrast the utility of topical immunosuppressants compared to systemic immunosuppressants in managing burn wounds. Topical steroids have demonstrated benefits to burn wound healing when specifically used in the context of preventing hypertrophic granulation tissue and unstable scar development [16]. Some researchers have found that certain topical immunosuppressants, such as infliximab, can be helpful in preventing the progression of burn wounds to a higher degree by stifling the inflammatory microenvironment responsible for necrotic tissue expansion in partial-thickness burn wounds [17]. Their studies suggest that via inhibition of local inflammatory mediators (e.g., IL-1β), as well as inhibition of tissue infiltration by macrophages, TNF-alpha modulators play an important role in improving burn wound outcomes when used in specific contexts. Taken with other studies demonstrating the efficacy of TNF-alpha monoclonal antibodies in the treatment of lupus nephritis, these particular biologics, when used topically, may potentially have a place in the management of burn wounds in the setting of SLE [18-19].

Though the case at hand involved a particularly convoluted hospital course, it serves to highlight the need for further investigation of treatment guidelines that address burn management in patients requiring concomitant rheumatologic disease treatment. This case adds to a small yet growing body of literature addressing the clinical presentation and challenges to the management of burn wounds in the setting of SLE. Burn management in our patient amid a severe exacerbation of her SLE requiring multiple immunosuppressive agents risked a non-healing burn. The presence of multiple, interrelated, complicated, and severe organ system pathologies necessitated a multidisciplinary team approach involving careful coordination between the departments of rheumatology, trauma and critical care, surgery, neurology, and nephrology, in addition to various ancillary services including physical therapy and rehabilitation, nutrition, pain management, and wound care. Our team’s treatment approach, as well as the patient’s healing process and response to treatment, may help elucidate an ill-defined algorithm of burn management in patients presenting with concomitant rheumatological disease exacerbation.

## Conclusions

This case adds to a small yet growing body of literature addressing the clinical presentation and management of burn wounds in the setting of SLE. Burn management in our patient with severe comorbid SLE complications, such as neuromyelitis optica spectrum disorder, was complicated and unclear, but adds to the algorithm of burn treatment with concomitant rheumatological disease exacerbation. While some degree of delayed wound healing should be anticipated in patients that are on immunosuppressive agents such as methylprednisolone, mycophenolate mofetil, and hydroxychloroquine, our case highlights the possibility of complete burn resolution via topical therapy alone even in the face of considerable immunosuppressive therapy. The decision against surgical intervention with skin grafting in light of the significant potential for post-operative wound decompensation secondary to our patient's pharmacotherapy requirements underscores the value of strong interdisciplinary teamwork in the management of burn wounds in patients with autoimmune diseases manifesting systemically. Further research should aim at balancing the risk of excision and grafting versus local wound care in patients on immunosuppressive regimens with careful consideration of the size and severity of wounds. In addition, it is essential for clinical investigators to address the paucity of evidence-based guidelines for the treatment of patients who present at the intersection of burn care and rheumatology.
